# Exercise-related sudden cardiac arrest in London: incidence, survival, and bystander response

**DOI:** 10.1186/1757-7241-22-S1-O3

**Published:** 2014-07-07

**Authors:** Melanie J Edwards, Rachael T Fothergill, Mark Whitbread

**Affiliations:** 1 Clinical Audit & Research Unit, London Ambulance Service NHS Trust, London, UK

## Background

The incidence of, and survival from, exercise-related sudden cardiac arrest (SCA) in the general population in the UK, and in London specifically, has not been investigated. The prevalence of factors likely to be related to survival from exercise-related SCA, such as bystander CPR and use of public defibrillators, has also not been examined.

## Method

The study utilised a retrospective observational design using data from a two-year period held on the London Ambulance Service’s cardiac arrest registry. Patient report forms for cardiac arrests where resuscitation was attempted, a cardiac origin was presumed, and in locations where exercise may have occurred were reviewed. A SCA was considered to be exercise-related if the arrest occurred during exercise or within an hour of exercising.

## Results

The incidence of exercise-related SCA in London was estimated to be 6.1 per million per population per year. The majority of cases were male and incidence increased from age 40. The most common activities engaged in at the time of SCA were running/jogging, football and cycling. Just under one third of patients survived to hospital discharge whilst survival in the Utstein group was 42%. Three-quarters of arrests were witnessed by a bystander. Bystander CPR was initiated in 62% of cases. Public defibrillators were available in 7% of arrests but only used in 4% of arrests.

## Discussion

Incidence of exercise-related SCA in the general population in London is rare. Survival following exercise-related SCA is considerably higher than for non-exercise related SCA. Increased survival in this group of patients may be due to the higher percentage of patients with an initial shockable rhythm, younger age, and increased bystander CPR. Survival may be improved further with increased availability of public defibrillators and education of those participating in sports or employed at leisure facilities regarding the importance of early CPR and defibrillation.

